# Lidocaine and Pinacidil Added to Blood *versus* Crystalloid Cardioplegic Solutions: Study in Isolated Hearts

**DOI:** 10.21470/1678-9741-2017-0244

**Published:** 2018

**Authors:** Helison Pereira do Carmo, Karla Reichert, Daniela Diógenes de Carvalho, Lindemberg da Mota Silveira-Filho, Karlos Vilarinho, Pedro Oliveira, Orlando Petrucci

**Affiliations:** 1 Universidade Estadual de Campinas (Unicamp), Campinas, SP, Brazil.

**Keywords:** Models, Animal, Cardioplegic Solutions, Myocardial Reperfusion Injury, Heart Arrest, Induced/Methods

## Abstract

**Objective:**

The present study aimed the functional recovery evaluation after long term of cardiac arrest induced by Custodiol (crystalloid-based) *versus* del Nido (blood-based) solutions, both added lidocaine and pinacidil as cardioplegic agents. Experiments were performed in isolated rat heart perfusion models.

**Methods:**

Male rat heart perfusions, according to Langendorff technique, were induced to cause 3 hours of cardiac arrest with a single dose. The hearts were assigned to one of the following three groups: (I) control; (II) Custodiol-LP; and (III) del Nido-LP. They were evaluated after ischemia throughout 90 minutes of reperfusion. Left ventricular contractility function was reported as percentage of recovery, expressed by developed pressure, maximum dP/dt, minimum dP/dt, and rate pressure product variables. In addition, coronary resistance and myocardial injury marker by alpha-fodrin degradation were also evaluated.

**Results:**

At 90 minutes of reperfusion, both solutions had superior left ventricular contractile recovery function than the control group. Del Nido-LP was superior to Custodiol-LP in maximum dP/dt (46%±8 *vs*. 67%±7, *P*<0.05) and minimum dP/dt (31%±4 *vs*. 51%±9, *P*<0.05) variables. Coronary resistance was lower in del Nido-LP group than in Custodiol-LP (395%±50 *vs*. 307%±13, *P*<0.05), as well as alpha-fodrin degradation, with lower levels in del Nido-LP group (*P*<0.05).

**Conclusion:**

Del Nido-LP cardioplegia showed higher functional recovery after 3 hours of ischemia. The analysis of alpha-fodrin degradation showed del Nido-LP solution provided greater protection against myocardial ischemia and reperfusion (IR) in this experimental model.

**Table:** 

Abbreviations, acronyms & symbols	
ATP	= Adenosine triphosphate
CEUA	= Ethics Committee on the Use of Animals
COBEA	= Brazilian Council for Animal Experimentation
CR	= Coronary resistance
IR	= Ischemia and reperfusion
LV	= Left ventricle
RPP	= Rate pressure product
SEM	= Standard error of mean
Unicamp	= Universidade Estadual de Campinas

## INTRODUCTION

Cardioplegic solution administration in open-heart surgeries has been the main strategy enable to antagonize the effects of global ischemia and reperfusion (IR). Cardioplegic arrest in combination with hypothermia corresponds to a standard method to reduce cardiac metabolism during the period of absence of oxygen and substrate, avoiding the collapse of contractile function after reperfusion^[^^1^^,^^2^^]^.

Despite being essential, this scientific field faces a major challenge in determining a single type of cardioplegic solution protocol amidst a variety of surgical procedures. A pharmacological approach to drugs that act as cardioplegic agents makes this puzzle even more difficult to understand. In the presence of cardioplegic agents, these solutions can block or open ion channels present in the sarcolemma, influencing on the ion dynamic between extra and intracellular means.

In the present study, two cardioplegic agents with distinct actions on ion channels were evaluated, followed by (I) pinacidil, a potassium channel opener, and (II) lidocaine, a sodium channel blocker. Rationally, two different types of solution, very eminent in clinical practice, were elected to drive these drugs on myocardium during global ischemia^[^^3^^]^.

Firstly, Custodiol solution (Custodiol^®^-HTK), commonly known as HTK solution (histidine/tryptophan/alpha-ketoglutarate) and which has been used primarily in organ preservation, was conceived as an alternative to crystalloid hyperkalemic cardioplegic solutions^[^^4^^,^^5^^]^. Custodiol solution has been used in some countries as the first choice for conventional cardiac surgeries, mainly in myocardial preservation^[^^6^^]^. It is considered a type of microplegia or cardioplegia solution with intracellular action due to reduction of potassium, sodium, and calcium concentration, resulting in cardiac arrest by polarization and avoiding damages from hypercontractures related to the depolarized arrest. In addition, Custodiol solution has an important buffering effect attributed to the amino acid histidine, which is important in controlling the balance of hydrogen ion levels released during a long period of ischemia^[^^7^^,^^8^^]^.

Secondly, del Nido cardioplegia, initially developed to be used in a single dose in neonatal surgeries, is classified as a modified hyperkalemic type due to high potassium concentration, although the lidocaine in its composition avoids the ionic imbalance by blocking sodium current flow^[^^9^^-^^11^^]^. This sodium channel blockage prevents intracellular calcium overload, avoiding contractile dysfunction after reperfusion^[^^11^^]^. Lidocaine is a selective and reversible blocker frequently used in clinical practice for the treatment of ventricular tachycardia and also has been assisted recovery hearts submitted to IR in experimental models^[^^12^^,^^13^^]^.

This study aimed to evaluate cardioplegic solutions under pharmacological modification. Pinacidil was added to both solutions and lidocaine only to the Custodiol solution, since del Nido cardioplegia original formulation already has this agent. Cardioplegic solutions were termed as Custodiol-LP and del Nido-LP. The main goal was to evaluate functional recovery after 3 hours of ischemia and 90 minutes of reperfusion. Moreover, cardiomyocyte integrity by alpha-fodrin degradation was also evaluated from heart samples collected at the end of the protocol.

## METHODS

### Animals

A total of 18 male Wistar rats weighing 250-350 g were used in this study, which was approved by the Ethics Committee on the Use of Animals (CEUA) of the Universidade Estadual de Campinas (Unicamp) and follows the ethic statements of the "Guide to the Care and Use of Laboratory Animals", published by the National Institute of Health of USA (NIH Publication No. 85-23, revised 1996), and the Brazilian Council for Animal Experimentation (COBEA).

### Surgical Protocol for Heart Perfusion

All animals received sodium thiopental anaesthesia (80 mg/kg) and sodium heparin (2,500 IU/kg) intraperitoneally. Then, a bilateral thoracotomy was performed exposing the heart to cardiectomy. The aorta was fixed to the system cannula with silk suture 4-0 and the perfusion was kept under constant pressure throughout the experiment. The catheter with latex balloon connected to the transducer pressure probe was inserted through the mitral valve and allocated to the left ventricle (LV). LV functional measurements of developed pressure, maximum dP/dt, minimum dP/dt, and rate pressure product (RPP) and the coronary resistance (CR) were done.

### Experimental Groups and Protocol

The hearts were assigned to one of the following three groups: control (n=4), Custodiol-LP (n=6) and del Nido-LP (n=6). They were initially perfused for 10 minutes stabilization to obtain baseline measurements. Cardiac arrest was induced with a single dose of cardioplegia (30 ml/kg), and the hearts were maintained in global ischemia at 4°C for 3 hours. Reperfusion was performed by re-establishing the heart perfusion.

### Cardioplegic Solutions Preparation

The Custodiol solution was acquired from a commercial vendor (Table 1). Blood cardioplegia del Nido was prepared with autologous blood collected directly from the thoracic cavity after heart explantation 1:4 (blood:solution) (Table 1).

**Table 1:** Composition of cardioplegic solutions.

Components (mM)	Custodiol-LP	del Nido-LP
Carrier	Sterile water	Plasma-Lyte A^®^
Blood:cardioplegia	__	1:4
Calcium chloride	0.02	0.24
Histidine	180	__
Histidine-HCl	18	__
Magnesium chloride	4	1.13
Magnesium sulfate	__	6.18
Mannitol	30	13.72
Potassium chloride	9	24.3
Potassium hydrogen 2-ketoglutarate	1	__
Sodium acetate trihydrate	__	20.34
Sodium bicarbonate	__	13.79
Sodium chloride	15	91.66
Sodium gluconate	__	17.33
Tryptophan	2	__
Drugs concentration (del Nido-LP without added blood)		
Lidocaine	0.45	0.45
Pinacidil	0.5	0.5

### Immunoblot by Western Blot Technique

The total protein extract with 50 µg per left ventricular sample was applied on 12% polyacrylamide gel (SDS-PAGE). Separation of the proteins of interest was performed by electrophoresis in mini-gel cell (Mini-PROTEAN II Cel® Bio-Rad) with buffer solution for electrophoresis previously diluted. Proteins were then transferred to a nitrocellulose membrane using electrotransfer equipment (Trans-Blot® Turbo(tm) Transfer System Bio-Rad). After transference, membranes were blocked with milk for subsequent incubation with primary alpha-fodrin (Abcam®, ab39165) at 4°C with constant stirring overnight (14-16 hours). These membranes were then washed with PBS solution and incubated in a chemiluminescent solution (Thermo Scientific Pierce ECL Western Blotting Substrate). Images were obtained by the multi-function system (Gel Logic Imaging System®, Rochester, NY, USA) to capture chemiluminescent reactions. All images were quantified by densitometry and normalized by colourimetric method with Ponceau^[^^14^^]^.

### Statistical Analysis

All data were expressed as percentage of recovery and as mean and standard error of mean (SEM). The hemodynamic data were normalized with baseline values. Analyses were performed by 2-way ANOVA with Sidak's *post-hoc* test for multiple comparisons. Comparison of protein expression was performed using non-parametric Student t-test. Statistical significance was assumed *P*<0.05. All statistical analyses were performed using GraphPad® Prism 6 software.

## RESULTS

### Functional Left Ventricle Analysis

**Fig. 1:**
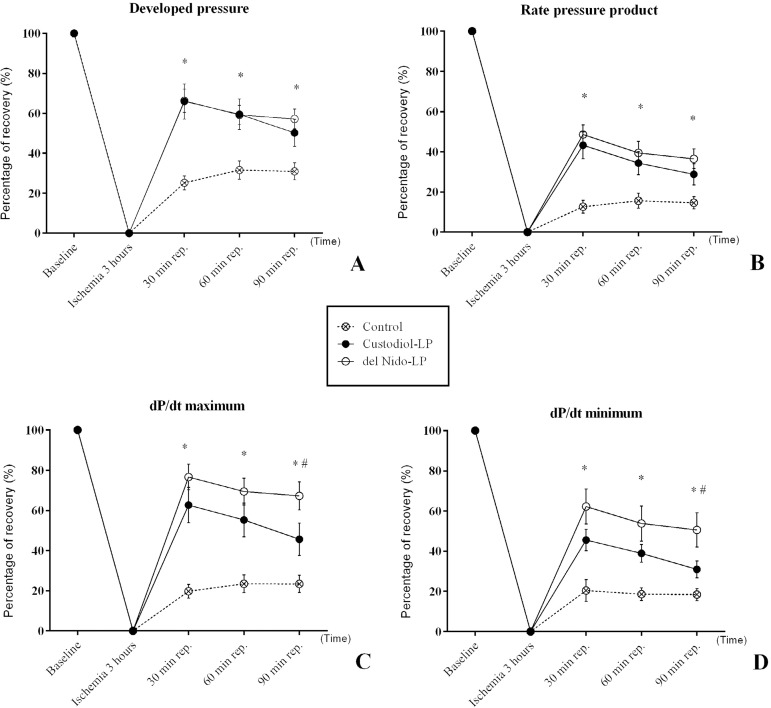
Left ventricular function data expressed as percentage of recovery after 3 hours. (A) Developed pressure. (B) Rate pressure product. (C) Maximum dP/dt. (D) Minimum dP/dt. All values are expressed as mean and standard error of mean (SEM) (error bars). Analyses were performed by 2-way ANOVA with Sidak's post-hoc test for multiple comparisons (*P < 0.05 compared to del Nido group). Six animals per group.

Two hearts from the control group were excluded due to ventricular fibrillation and no beating recovery after ischemia. Lower levels of developed pressure (Figure 1A) and RPP (Figure 1B) recovery were found in the control group than in Custodiol-LP and del Nido-LP groups, respectively. However, no relevant differences among the cardioplegic solution groups were found with these variables. Relevant lower levels of maximum dP/dt (Figure 1C) and minimum dP/dt (Figure 1D) were found in the control group than in Custodiol-LP and del Nido-LP groups, respectively. However, at 90 minutes of reperfusion, higher levels of maximum dP/dt (Figure 1C) and minimum dP/dt (Figure 1D) were found in del Nido-LP group than in Custodiol-LP.

### Coronary Resistance

**Fig. 2:**
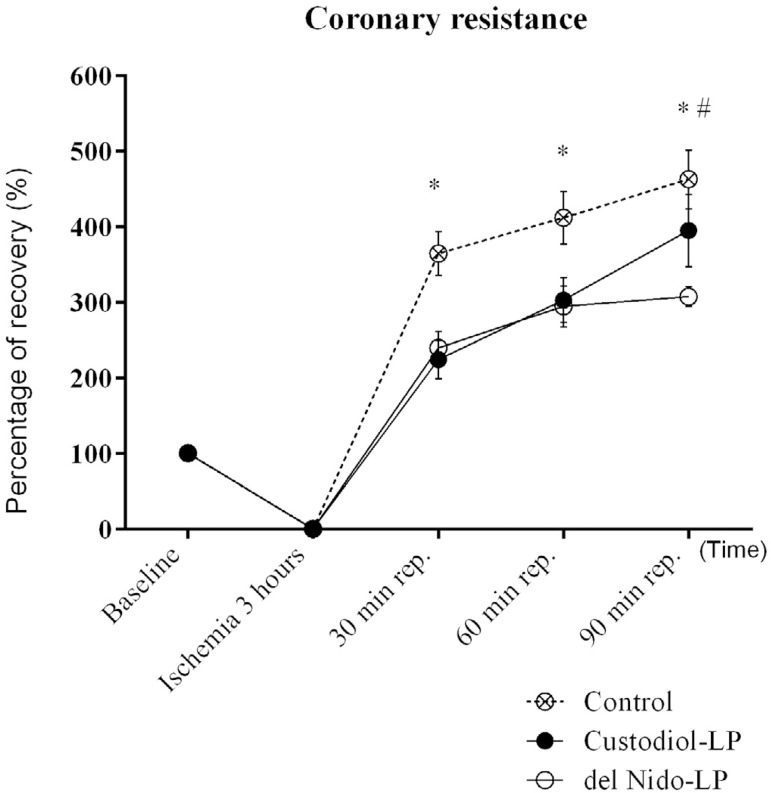
Coronary resistance data expressed as percentage of recovery after 3 hours. All values are expressed as mean and standard error of mean (SEM) (error bars). Analyses were performed by 2-way ANOVA with Sidak's post-hoc test for multiple comparisons (*P<0.05 compared to del Nido group). Six animals per group.

CR levels found in the control group were higher than in Custodiol-LP group up to 60 minutes of reperfusion (Figure 2). Compared to del Nido-LP group, the control group showed high levels of CR throughout reperfusion. However, del Nido-LP group showed lower levels of CR than Custodiol-LP group at 90 minutes of reperfusion (Figure 2).

### Protein Expression by Western Blot Technique

**Fig. 3:**
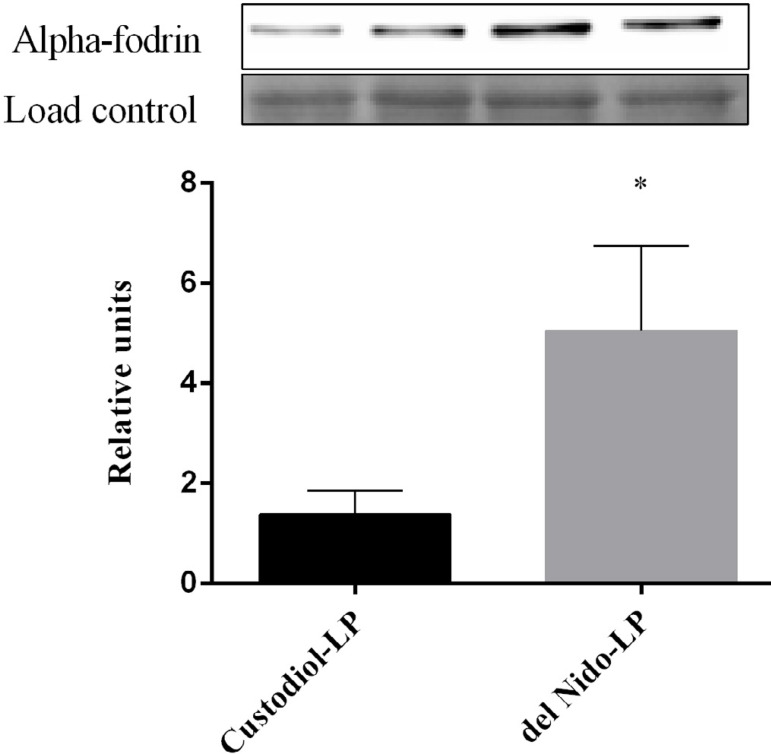
Representative Western blot results for alpha-fodrin. All values are expressed as mean, standard error of mean (SEM) (error bars), and arbitrary units. Analyses were performed by t-test (*P<0.05). Four animals per group.

Higher degradation of alpha-fodrin expression was found in Custodiol-LP group than in del Nido group in samples collected after 3 hours of ischemia and 90 minutes of reperfusion (Figure 3).

## DISCUSSION

Myocardial global IR insult is well known to promote multifactorial effects on cellular metabolism resulting in irreversible damage to the heart function. Cardioplegic solutions with pharmacological combination as an alternative to provide further myocardial protection have encouraged researchers to carry out different combinations^[^^15^^]^. Therefore, the present study evaluated pharmacological modifications with sodium inhibitor channels and potassium channel openers added to clinical relevant cardioplegic solutions, Custodiol and del Nido.

The potassium channel opener pinacidil has two main effects: vasodilatation and contractility reduction, due to its effect on potassium efflux avoiding calcium overload into the cardiomyocytes^[^^16^^]^. In experimental models, pinacidil induced cardiac arrest by polarization, consequently reducing the adenosine triphosphate (ATP) consumption, compared to the cardiac arrest induced by depolarization^[^^17^^,^^18^^]^. Pinacidil has a non-selective pharmacological effect regarding the potassium channels' location, being able to activate them in the sarcolemma and mitochondria, since both have an important role in myocardial protection^[^^17^^,^^19^^]^. Differently, lidocaine is a reversible sodium channel blocker which has a pharmacological effect on calcium overload, preventing sodium-calcium exchanger reverse-mode action during IR^[^^9^^,^^10^^]^. In combination, these cardioplegic agents might improve cardiac resistance against long-term ischemia.

Custodiol-LP showed detrimental effects on cardiac function by the maximum and minimum dP/dt at 90 minutes of reperfusion compared to del Nido-LP. However, developed pressure and RPP parameters were similar throughout reperfusion time. CR analysis showed Custodiol-LP group with higher levels than del Nido-LP and marked reduction of coronary flow after 90 minutes of reperfusion.

The superiority of del Nido-LP solution may be associated with its greater ability to protect the myocardium over prolonged ischemia. The immunoblot assay performed by Western blot technique showed alpha-fodrin significant degradation in Custodiol-LP group compared to del Nido-LP. Alpha-fodrin present in extracellular matrix is consumed as a substrate for calpains after IR. The increase of calpain activity is correlated with myocardial injury and loss of contractile function^[^^20^^-^^22^^]^. Therefore, alpha-fodrin degradation is an indirect marker for cell integrity.

According to our findings, the addition of pharmacological agents to cardioplegic solutions showed greater myocardial protection in del Nido-LP group than in Custodiol-LP. Other components present in this solution may have possibly collaborated for these results. A single dose of the del Nido original formulation per se has shown great efficacy in pediatric and adult surgeries; perhaps, new solution proposals to induce long periods of ischemia should be based on its composition.

## CONCLUSION

The addition of cardioplegic agents pinacidil and lidocaine provided greater protective capacity to del Nido-LP solution than to Custodiol-LP in hearts submitted to 3 hours of ischemia and 90 minutes of reperfusion. Contractile function data, according to variables of maximum dP/dt, minimum dP/dt and CR, showed a higher percentage of recovery in del Nido-LP group. Also, lower levels of alpha-fodrin degradation in del Nido-LP can support these findings showing higher myocardial protection against IR injury.

**Table:** 

Authors' roles & responsibilities	
HPC	Substantial contributions to the conception or design of the work; or the acquisition, analysis, or interpretation of data for the work; final approval of the version to be published
KR	Substantial contributions to the conception or design of the work; or the acquisition, analysis, or interpretation of data for the work; final approval of the version to be published
DDC	Substantial contributions to the conception or design of the work; or the acquisition, analysis, or interpretation of data for the work; final approval of the version to be published
LMSF	Substantial contributions to the conception or design of the work; or the acquisition, analysis, or interpretation of data for the work; final approval of the version to be published
KV	Substantial contributions to the conception or design of the work; or the acquisition, analysis, or interpretation of data for the work; final approval of the version to be published
PO	Substantial contributions to the conception or design of the work; or the acquisition, analysis, or interpretation of data for the work; final approval of the version to be published
OP	Substantial contributions to the conception or design of the work; or the acquisition, analysis, or interpretation of data for the work; final approval of the version to be published
